# Novel Use of Fresh Frozen Plasma in Treating Hereditary Angioedema: A Success Story From Pakistan

**DOI:** 10.7759/cureus.9669

**Published:** 2020-08-11

**Authors:** Amber Sabeen Ahmed, Sidra Fayyaz

**Affiliations:** 1 Medicine, Aga Khan University Hospital, Karachi, PAK; 2 Pulmonology, Aga Khan University Hospital, Karachi, PAK

**Keywords:** hereditary angioedema, fresh frozen plasma, intensive care

## Abstract

Hereditary angioedema (HAE) due to a C1-esterase inhibitor(C1-INH) deficiency is a rare and potentially life-threatening disorder. It is characterized by an episodic and self-limiting increase in vascular permeability. The condition manifests itself as recurrent attacks of swelling in any part of the body. The angioedema can cause the involvement of the respiratory tract, skin, and gastrointestinal tract. Laryngeal involvement can make the condition life-threatening.

It does not respond well to conventional angioedema therapy of steroids, adrenaline, and antihistamines. The targeted therapy for HAE consists of plasma-derived or recombinant C1-INH, ecallantide, and icatibant or bradykinin receptor antagonist. In the absence of these therapies, it becomes difficult to manage this condition effectively. We present a case of hereditary angioedema, who presented with life-threatening laryngeal edema, causing asphyxia, leading to cardiac arrest. Due to a lack of availability of C1-INH concentrate, he was given fresh frozen plasma (FFP). His condition gradually improved, and he was successfully extubated after three days. This is the first time we are reporting a case from Pakistan in which the patient was successfully treated with FFP for an acute attack of hereditary angioedema.

## Introduction

Hereditary angioedema due to C1-esterase inhibitor(C1-INH) deficiency is a rare, life-threatening disease that affects about one in 10,000 to one in 150,000 people [1]. Seventy-five percent of cases are autosomal dominant and 25% of cases are due to spontaneous mutation. It is caused by an inherited deficiency of C1-INH that is either quantitative (type 1) [2-3] or functional (type 2) [4].

C1-INH is a key inhibitor of factor XIIa, factor XIIf, and plasma kallikrein, all of which are important enzymes in the kallikrein-kinin cascade. The deficiency of C1-INH causes the activation of the cascade. The high molecular weight kininogen, especially, has excessive cleavage, which causes increased bradykinin production [5]. The bradykinin thus produced causes increased vascular permeability, which, in turn, causes swelling and inflammation.

Serum C4 level is a readily available screening test for HAE, with almost always decreased levels during attacks.

Supportive treatment with antihistamines and corticosteroids has a very limited role in its management. The mainstay of emergency treatment in acute attacks includes C1-INH concentrate (plasma-derived or recombinant) [6-7] or bradykinin-receptor antagonists. The targeted therapies are, however, not available in most low to middle-income countries (LMICs) like Pakistan. There is some published data about the use of fresh frozen plasma (FFP) for acute treatment of the condition [8]. We present here a case of life-threatening, hereditary angioedema due to a C1-esterase inhibitor deficiency being successfully managed with fresh frozen plasma.

## Case presentation

A 30-year, single male, accountant by occupation, presented to the emergency department of Aga Khan University Hospital, with severe bronchospasm, respiratory distress, and angioedema. His symptoms developed, following a fall on the ground with a minor injury to his face, over the area below left lower eyelid, six hours back. The swelling started over the area of trauma and gradually spread all over the face, including both eyelids. He had no history of ingestion of any food or medicines prior to the development of swelling. He had a history of similar but mild multiple attacks in the past (eight to 10 times in the last three years).

On arrival to the emergency room (ER), he was in obvious respiratory distress with an audible wheeze and hoarse voice. He was tachypneic with a respiratory rate of 38/min and had a heart rate of 135/min with a blood pressure of 100/75. He was given intravenous (IV) steroids, adrenaline, and antihistamine immediately, with no relief of his symptoms. His condition worsened, and he suddenly went into cardiorespiratory arrest. He had cardiopulmonary resuscitation (CPR) of around five mins with pulseless electrical activity (PEA) and was intubated and shifted to the intensive care unit (ICU). His medical records showed him to be a case of hereditary angioedema. He was started on fresh frozen plasma (FFP) after two hours of ER admission, as there was no other treatment option available. He continued receiving FFP every six hours until the resolution of his symptoms, which took around 18 hours. His laryngeal edema resolved suggested by a positive cuff leak, which was detected on deflating the ballon of his endotracheal tube. He didn’t develop any adverse reactions related to transfusion. He was successfully extubated on Day 3 of his ICU stay. His blood sample, taken at the time of intubation, before any FFP transfusion, showed a low C4 level 0.10 g/l (normal range (0.12-0.36)). The patient recovered fully and was shifted to a monitored step-down unit and then a general care ward. The patient and family members were educated regarding the avoidance of triggering factors and early reporting to the hospital if the symptoms develop. He was later discharged from the hospital on Day 5 to be followed in the clinic and was prescribed danazol for prophylaxis.

## Discussion

Our case describes the first published successful management of life-threatening HAE with FFP from Pakistan. HAE, first described in 1882, is an autosomal dominant hereditary disorder [9]. Clinically, HAE presents as recurrent bouts of nonpruritic subcutaneous and submucosal swelling, which can be localized to the extremities, face, bowels, or upper respiratory tract. Abdominal episodes may occur, accompanied by severe nausea and pain mimicking an acute abdomen.

Laryngeal edema, if present, can significantly contribute to mortality, which is reported at around 15%-30% in the literature [3]. Our patient developed significant laryngeal edema leading to significant hypoxia and cardiopulmonary arrest. Based on his previous diagnosis of HAE, our patient was started on FFP.

The recommended treatment of this life-threatening complication of HAE is C1 inhibitor (C1-INH) concentrates (plasma-derived or recombinant) [5-6] or bradykinin-receptor antagonists. Ecallantide, which is a plasma kallikrein inhibitor, and icatibant, a bradykinin B2 receptor antagonist, have been recently approved by the Food and Drug Administration (FDA) for the management of acute attacks of HAE [8].

In the absence of these specific therapies in Pakistan, FFP remained the only treatment option available for our patient, as it contains C1-INH [10-11]. This therapy was started two hours after ER arrival, which was also the median time noted in a detailed study from Africa and Iran, analyzing a few episodes of HAE [11].

The dosage of FFP received by our patient was 6 units (approx.20 ml/kg), which led to a complete resolution of symptoms in 18 hours. There is limited published data about the exact dosage of FFP. In a few studies, estimates of 1-4 units or 20 mL/kg of FFP were found to be effective [11-12].

Although HAE literature suggests occasional side effects with its use, we did not encounter any adverse effects. There are also concerns about its safety in some studies, suggesting that its use can precipitate angioedema due to harmful substrates [13]. 

FFP treatment offered in a timely manner to our patient resulted in the complete resolution of angioedema and was a lifesaver. Alloimmunization and volume overload, known as the adverse effects of FFP usage, were also not observed in our patient. In our resource-limited setting, with the unavailability of targeted therapies, FFP treatment proved itself to be highly effective and safe in the management of our patient.

## Conclusions

This is the first time the successful treatment of hereditary angioedema with FFP has been reported from Pakistan. In the absence of targeted therapy in most LMICs, FFP is an alternative therapeutic option for the acute management of HAE. We need comparative studies between targeted therapies and FFP to establish its use for this rare condition, especially in resource-poor settings. We also need to have clear recommendations for FFP use, regarding treatment thresholds, time to start therapy, and dosing.
